# Development and Application of Real-Time PCR Assay for Detection of Salmonella Abortusequi

**DOI:** 10.1128/jcm.01375-22

**Published:** 2023-03-01

**Authors:** Jinhui Wang, Kui Guo, Shuaijie Li, Diqiu Liu, Xiaoyu Chu, Yaoxin Wang, Wei Guo, Cheng Du, Xiaojun Wang, Zhe Hu

**Affiliations:** a State Key Laboratory of Veterinary Biotechnology, Harbin Veterinary Research Institute, Chinese Academy of Agricultural Sciences, Harbin, China; Marquette University

**Keywords:** *Salmonella* Abortusequi, real-time PCR, FljB, clinical detection, equine, donkey

## Abstract

Salmonella enterica subsp. *enterica* serovar Abortusequi is a major pathogen in horse and donkey herds, causing abortion in pregnant equids and resulting in enormous economic losses. A rapid and reliable method is urgently needed to detect *S*. Abortusequi in herds where the disease is suspected. To achieve this goal, a TaqMan-based real-time PCR assay targeting the gene for the flagellin protein phase 2 antigen FljB was developed. This real-time PCR assay had high specificity, sensitivity, and reproducibility. The detection limit of the assay was 30 copies/μL of standard plasmid and 10 CFU/μL of bacterial DNA. Furthermore, 540 clinical samples, including 162 tissue, 192 plasma, and 186 vaginal swab samples collected between 2018 and 2021 in China, were tested to assess the performance of the developed assay. Compared to the gold standard method of bacterial isolation, the real-time PCR assay exhibited 100% positive agreement for all tissue, plasma and vaginal swab tests. Additionally, this assay detected DNA from *S.* Abortusequi from 56.7% (34/60) culture-negative tissue and 22.9% (41/179) culture-negative vaginal swab samples from infected equids. Receiver operating characteristic analysis demonstrated that the results of the developed real-time PCR assays were in significant agreement with those of the culture method. The real-time PCR assay can be completed within 45 min of extraction of DNA from samples. Our results show that this assay could serve as a reliable tool for the rapid detection of *S.* Abortusequi in tissue, plasma, and vaginal swab clinical samples.

## INTRODUCTION

Salmonella is a significant foodborne pathogen that causes gastroenteritis, primary bacteremia, and enteric fever in severe cases (1–3). Two species are currently known from the genus Salmonella, Salmonella enterica and Salmonella bongori (4), and more than 2,600 Salmonella serovars belonging to S. enterica have been identified (5). S. enterica can be subdivided into six subspecies, with only the first subspecies known to infect warm-blooded animals (1, 6). Salmonella enterica subsp. *enterica* serovar Abortusequi is the causative agent of equine abortus salmonellosis, which is characterized by abortion in pregnant equids (7, 8). Horses and donkeys are the main hosts of *S.* Abortusequi (9, 10). Since the 1980s, there have been few reports worldwide of equine abortus salmonellosis, resulting in scientific neglect of the disease. However, in China, unexpectedly, the disease has reemerged, initially only with very small numbers of cases, but the numbers have been increasing gradually over the last decade.

Infection with *S.* Abortusequi can cause abortion in horses in the late stages of pregnancy, as well as death in newborn foals and arthritis in young foals (11). The aborted fetus, fetal membranes, and amniotic fluid are infectious, and the disease can be transmitted vertically to the newborn fetus and horizontally to other horses (8). Clinical observations show that pregnant mares abort suddenly and without any prior symptoms, and mares who have had an abortion may abort again due to infection with *S.* Abortusequi. In farm environments, *S.* Abortusequi is resistant to many specific drugs and cannot be completely eradicated from the environment. The infection causes huge economic losses in equine husbandry, mainly because of the growing number of abortions. An efficient method to detect *S.* Abortusequi expeditiously is fundamental for the implementation of disease prevention strategies.

Conventional Salmonella detection methods involve the isolation of bacteria, biochemical identification of presumptive colonies, and serotyping of Salmonella, which identifies lipopolysaccharide (O antigen) and flagellin (H antigen), according to the Kauffmann-White-Le Minor scheme (12, 13). However, the complexity of the scheme brings a number of drawbacks: it is labor-intensive, time-consuming, and expensive (14–16). Typically, completion of the entire process requires at least 3 days (17). Moreover, a series of different typing antisera and antigens are available for serotype identification, and these are expensive and must be ordered to complete the tests. Over the past 2 decades, real-time PCR, which allows high throughput and is both highly sensitive and strongly specific, has been frequently applied as a potential method for the testing of Salmonella (18–20). To date, several real-time PCRs have been developed using different genes. The STM4200 and SEN1392 genes have been used for the differentiation of S. enterica serovar Typhimurium and S. enterica serovar Enteritidis (21), and the *invA* gene has been used for the detection of Salmonella (20).

*S.* Abortusequi is a common causative agent of equine abortus salmonellosis, but it is not the only serovar reported as being able to cause this condition, with *S.* Typhimurium (group B), S. Enteritidis and *S.* Dublin (group D) also causing this disease in equids in rare cases (22). However, the primers used in the real-time PCR tests mentioned above were not able to distinguish *S.* Abortusequi from the other serovars, and the development of a specific PCR assay for the detection of *S.* Abortusequi is therefore of great importance.

Almost all Salmonella species are flagellate, with the two exceptions being *S.* Pullorum and *S.* Gallinarum. Most serovars of Salmonella are biphasic strains; for example, *S.* Typhimurium contains two different flagellin genes, FliC and FljB. However, *S.* Dublin only expresses the phase 1 flagellar antigen encoded by FliC and, similarly, *S.* Abortusequi lacks the phase 1 flagellar antigen and only expresses the phase 2 flagellar antigen encoded by FljB. The flagellin genes are about 1,500 bp in length and comprise two conserved terminal regions as well as a highly variable central region that differs between different Salmonella serotypes (23). The flagellin gene is commonly used as a serotype detection identifier for Salmonella (24, 25), and the phase 2 flagellar FljB gene can be considered a good candidate gene for the detection of *S.* Abortusequi.

In this study, the nucleotide sequences of the FljB and/or FliC genes from different Salmonella serotypes and other members of *Enterobacterales* were aligned to determine any sequence fragments specific to *S.* Abortusequi that would allow for the accurate diagnosis of the cause of equine abortion. We then developed a sensitive and specific real-time PCR assay for the detection of *S.* Abortusequi targeting the FljB gene. We evaluated the assay using clinical samples of tissue, plasma, and vaginal swabs, and we compared the results to reference culture methods. Here, to our knowledge, is the first report on the development and application of a real-time PCR assay for the detection of *S.* Abortusequi.

## MATERIALS AND METHODS

### Bacteria and virus strains.

The *S.* Abortusequi (AES strain) used in this study were isolated by our laboratory from tissue of aborted foals in clinical trial. The other three Salmonella strains used (*S.* Typhimurium, S. Enteritidis, and *S.* Dublin) were the kind gift of Guoqiang Zhu at the college of Veterinary Medicine, Yangzhou University (Yangzhou, Jiangsu, China). Non-Salmonella pathogens used in this study included equine influence virus (EIV), equine herpesvirus-1 (EHV-1), equine herpesvirus-4 (EHV-4), equine arteritis virus (EAV), Streptococcus equi subsp. *equi*, Escherichia coli, and equine infectious anemia virus (EIAV) and were taken from laboratory stocks. The strains listed above were used to analyze the specificity of the *S.* Abortusequi real-time PCR assay.

### Clinical samples.

A total of 540 clinical samples, including 162 tissue, 192 plasma, and 186 vaginal swab samples from either aborted fetuses or adult equids, were collected from 14 farms located across Inner Mongolia, Shan Dong, Hebei, Gansu and Heilongjiang Provinces, between 2018 and 2021.

For pathogen culture, 50 μL of plasma or vaginal swab liquid or one loopful of tissue was plated on chromogenic Salmonella agar medium (Chromagar, France) and incubated at 37°C overnight. Suspicious purple colonies were identified using Salmonella typing antisera sets for typing of the O-antigen (Meizheng Biotech, China) and H-antigen (Statens Serum Institut, Denmark) based on the slide agglutination reaction. To avoid cross-reaction with other Enterobacter species, 16S rRNA genes from all isolates were amplified and sequenced. When the antigenic formula of the isolate serotyped was found to be 4,12:-:e,n,x, and the result of 16S rRNA gene sequencing was determined to be Salmonella, the isolate was determined to be *S.* Abortusequi.

DNA was extracted from all kinds of clinical samples using a TIANamp genomic DNA kit (Tiangen, China) according to the manufacturer’s instructions. A volume of 1 mL bacterial suspension of *S.* Abortusequi at an initial concentration of 10^8^ CFU/mL was extracted and eluted in 100 μL of Tris-EDTA buffer. Bacterial DNA at a concentration of 10^7^ CFU/μL was then generated. A 2-μL aliquot of DNA was used as the template in the *S.* Abortusequi real-time PCR assay.

### *S.* Abortusequi real-time PCR assay.

**FIG 1:**
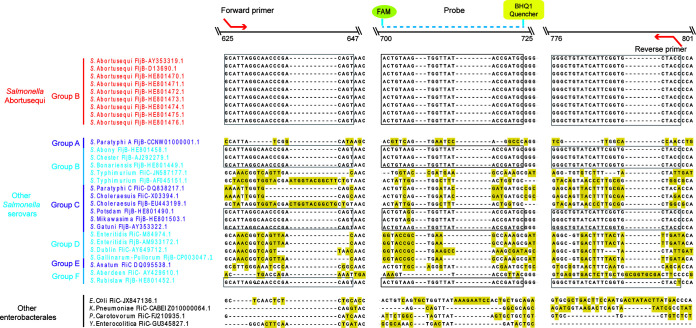
Sequence alignment of *fljB* and/or *fliC* genes from *S.* Abortusequi, other Salmonella serotypes, and other members of *Enterobacterales* over the positions of probe and primers.

All available FljB and/or FliC genes from *S.* Abortusequi, other Salmonella serovars (groups A to F), and other members of *Enterobacterales* were aligned using the Megalign software (DNAStar, USA). The specific primers and probe were designed for *S.* Abortusequi using the Primer 5.0 software (Bio Tools, USA) (Fig. 1). The sequences of the primers and probe for the *S.* Abortusequi FljB gene were as follows: FljB-F, 5′-CATTAGGCAACCCGACAGTA-3′; FljB-R, 5′-GGTAGCACCGAATGATACAGC-3′; FljB-P, 5′–6-carboxyfluorescein–ACTGTAAGTGGTTATACCGATGC–MGB–3′. Real-time PCR tests were carried out in 20-μL reaction mixtures using FastFire qPCR premix probe (Tiangen, China). Briefly, reaction mixtures comprised 10 μL 2× FastFire qPCR premix, 0.6 μL of each primer (10 μM), 0.4 μL of probe (10 μM), 2 μL of bacterial DNA, and 6.4 μL of double-distilled water (ddH_2_O). The amplification was performed on a QuantStudio 5 system (Thermo Fisher Scientific, USA). The cycling parameters were 95°C for 1 min, followed by 40 cycles of 95°C for 5 s and 60°C for 15 s. The *S.* Abortusequi-positive samples identified with the real-time PCR assay were confirmed by sequencing, a process which will not be included in routine detection.

### Construction of the standard curve.

In order to construct the standard plasmid, the FljB gene was amplified and cloned into a pMD18T vector. The standard plasmid was then transformed into DH5α chemically competent cells (Tiangen, China) and purified with a FastPure Gel DNA extraction minikit (Vazyme, China) according to the manufacturer’s instructions. The concentration of the purified plasmid DNA was determined by measuring the optical density at 260 nm (OD_260_) with a NanoPhotometer P330 (Berthold, Germany). The copy number of the plasmid was calculated according to the following formula: Y (copies per microliter) = [(6.02 × 10^23^) × plasmid DNA concentration (in grams per microliter)]/[plasmid DNA length × 660]. The standard curve was constructed from the amplification of serial 10-fold dilutions of the standard plasmid ranging from 3 × 10^7^ to 3 × 10^0^ copies/μL.

### Universal internal positive control.

A universal internal positive control (IPC) described previously (26) was used to determine the PCR inhibitors in the assay. The IPC plasmid was synthesized, including the sequence fragment of the IPC by BGI Tech Solutions (Beijing Liuhe, China). The sequences of primers and probe and reaction system were the same as described earlier (26). One thousand copies of IPC plasmid were used in each sample preparation.

### Analysis specificity, sensitivity and repeatability.

The analysis specificity was evaluated by testing the nucleic acid extracted from *S.* Abortusequi, S. equi, EIV, EHV-1, EHV-4, EIAV, EAV, E. coli, *S.* Typhimurium, S. Enteritidis, and *S.* Dublin. The analysis sensitivity was assessed by testing serial 10-fold dilutions of the standard plasmid (3 × 10^7^ to 3 × 10^0^ copies/μL) and the bacterial DNA (1 × 10^6^ to 1 × 10^0^ CFU/μL) in triplicate. Serial dilutions of the standard plasmid (3 × 10^2^, 3 × 10^1^, and 3 × 10^0^ copies/μL) and of the bacterial DNA (1 × 10^2^, 1 × 10^1^, and 1 × 10^0^ CFU/μL) were tested in 10 repetitions to determine the detection limit, where the detection rate of 10 repetitions was 100%. For intra-assay repeatability, each dilution of the standard plasmid (3 × 10^5^ to 3 × 10^0^ copies/μL) and the bacterial DNA (1 × 10^5^ to 1 × 10^0^ CFU/μL) was tested three times in one run. For interassay repeatability, each dilution was tested in triplicate in three independent experiments on three different days.

### Statistical analysis.

To evaluate the diagnostic value of the *S.* Abortusequi real-time PCR assay, a receiver operating characteristic (ROC) analysis was constructed in GraphPad Prism8 (GraphPad Software, USA) using the culture as gold standard. The optical cutoff was determined using the ROC curve. The Wilson-Brown test was used to determine the confidence intervals. The sensitivity and specificity of the assay to different types of samples were calculated at the 95% CI level.

## RESULTS

### Design of primers and probe for real-time PCR based on the *fljB* gene of *S*. Abortusequi.

In total, 32 nucleotide sequences of the *fljB* and/or *fliC* genes from 6 Salmonella serotypes and other members of *Enterobacterales* were aligned, and the *fljB* genes were found to be conserved among all 9 different strains of *S.* Abortusequi. The primers and probe were designed for real-time PCR to amplify the fragment of the FljB gene with a length of 174 bp. This design can distinguish *S.* Abortusequi from *S.* Typhimurium, S. Enteritidis, *S.* Dublin, and most Salmonella serotypes from group A to group F (Fig. 1). It is worth noting that the primers also matched the *fljB* gene of seven strains from other Salmonella serovars. However, these other Salmonella serovars have never been reported to cause miscarriages in horses.

### Standard curve of the *S.* Abortusequi real-time PCR assay.

The standard curve of the *S.* Abortusequi real-time PCR was constructed from the amplification of serial 10-fold dilutions of the standard plasmid ranging from 3 × 10^7^ to 3 × 10^0^ copies/mL. The curve showed a high linearity, with a correlation coefficient (*R*^2^) of 0.999. The curve had a slope of −3.271, an intercept of 37.273, and thus a formula of *y* = −3.271*x* + 37.273 (see Fig. S1A in the supplemental material). The amplification efficiency of the *S.* Abortusequi real-time PCR was calculated to be 102.183%.

### Analytical specificity of the *S.* Abortusequi real-time PCR assay.

We assessed the specificity of our *S*. Abortusequi real-time PCR assay using nucleic acids from S. equi, EIV, EHV-1, EHV-4, EIAV, EAV, and E. coli, which represented the most common infectious pathogens in horses, and from *S.* Typhimurium, S. Enteritidis and *S.* Dublin, which represented the other three possible causative agents of equine abortions due to Salmonella. The DNA of *S.* Abortusequi and DNase-free dH_2_O were amplified as positive and negative controls, respectively. Only the *S.* Abortusequi template resulted in an amplification curve in this assay, and no fluorescence signal was obtained from other templates. These results suggest that the *S*. Abortusequi real-time PCR assay is specific to *S.* Abortusequi (Fig. S1B).

### Analytical sensitivity of the *S.* Abortusequi real-time PCR assay.

The preliminary assessment for analytical sensitivity of the *S.* Abortusequi real-time PCR assay was performed with 10-fold dilutions of the standard plasmid and *S.* Abortusequi bacterial DNA. Amplification curves were observed in all three replicates when the concentration of the standard plasmid was ≥ 3 × 10^0^ copies/μL and that of the bacteria was ≥ 1 CFU/μL (Fig. S1C and D). Serial dilutions of the standard plasmid (3 × 10^2^, 3 × 10^1^, and 3 × 10^0^ copies/μL) and of the bacterial DNA (1 × 10^2^, 1 × 10^1^, and 1 × 10^0^ CFU/μL) were tested in 10 repetitions for further confirmation of the sensitivity. Concentrations of 3 × 10^2^ and 3 × 10^1^ copies/μL of the standard plasmid and of 1 × 10^2^ and 1 × 10^1^ CFU/μL of the bacterial DNA tested positive in all 10 repetitions. For 3 × 10^0^ copies/μL of standard plasmid and 1 × 10^0^ CFU/μL of bacterial DNA, the detection rates were 80% (8/10) and 30% (3/10), respectively (Table S1). Therefore, the detection limit of the assay was 30 copies/μL of the standard plasmid and 10 CFU/μL of bacterial DNA.

### Reproducibility of the *S*. Abortusequi real-time PCR assay.

The reproducibility of the *S*. Abortusequi real-time PCR assay was evaluated using 10-fold dilutions of the standard plasmid and of *S.* Abortusequi bacteria DNA. The coefficients of variation (CV) for intra-assay and interassay were less than 5% at all dilutions, indicating good reproducibility of the assay (Table S2).

### The *S.* Abortusequi real-time PCR assay showed little interference from PCR inhibitors.

**FIG 2:**
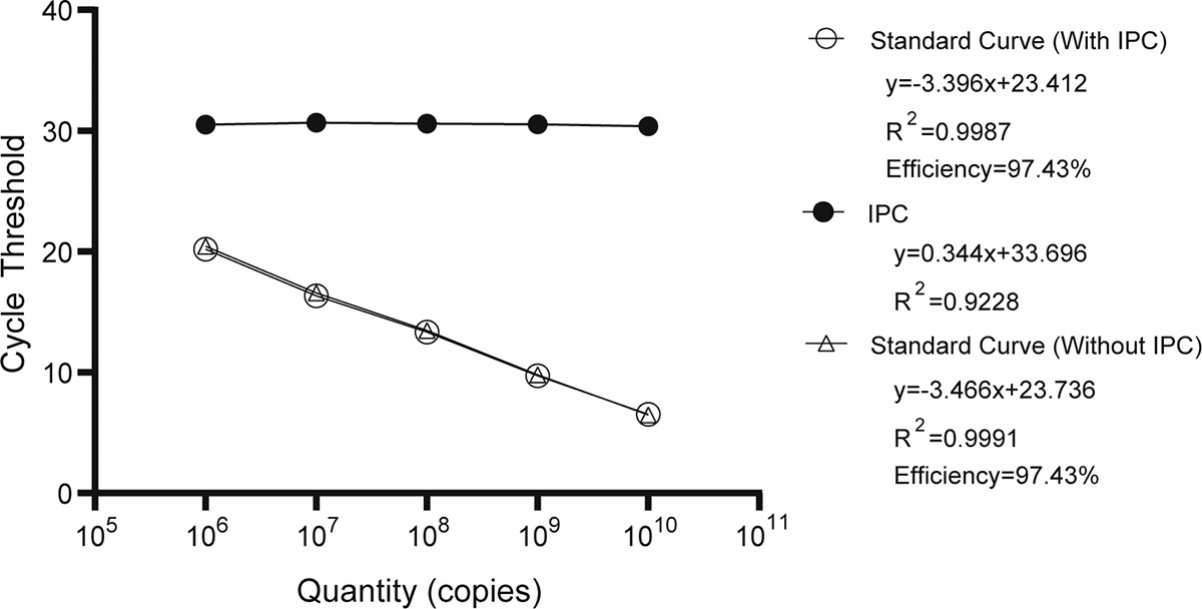
*S.* Abortusequi real-time PCRs with or without IPC. Ten-fold dilutions of the standard plasmid were tested alone or along with an internal positive control (1,000 copies) in triplicate by using *S.* Abortusequi real-time PCRs. The amplification efficiencies were calculated from the slopes of the linear regressions.

To evaluate the possible influence caused by PCR inhibitors during the PCR assay, an IPC was used in the standard curve assay and clinical sample detection. For testing the different concentrations of standard plasmids, the threshold cycle (*C_T_*) value for the *fljB* gene and the resulting amplification efficiencies had few differences in *S*. Abortusequi real-time PCR assays with and without IPC (Fig. 2). In a test with 5 tissue samples obtained from aborted foals, the CVs for the *C_T_* value for IPC was only 1.26% between *S*. Abortusequi real-time PCR with IPC addition and the real-time PCR with IPC alone (Table S3). These results suggested that there were no PCR inhibitors in the samples from aborted foals for the *S*. Abortusequi real-time PCR.

### Performance of the *S.* Abortusequi real-time PCR assay on clinical samples.

The 162 tissue, 192 plasma, and 186 vaginal swab samples, which were collected from horses and donkeys between 2018 and 2021, were tested concurrently using our *S*. Abortusequi real-time PCR assay and the culture method. Of the 162 tissue, 192 plasma, and 186 vaginal swab samples tested using the *S*. Abortusequi real-time PCR assay, 102 tissue, 2 plasma, and 7 vaginal swab samples were identified as positive, which was in 100% agreement with the reference culture method. The negative results were from 26 tissue samples, whereas 190 plasma samples and 138 vaginal swab samples were also in agreement with the reference culture method. Moreover, *S.* Abortusequi DNA was detected in 34 culture-negative tissue samples and 41 culture-negative vaginal swab samples by our assay. The resulting amplicons were cloned and sequenced, confirming that these sequences were indeed from *S.* Abortusequi. The detection rate of tissue, plasma, and vaginal swab samples using the *S*. Abortusequi real-time PCR assay was 83.95%, 1.04%, and 25.81% (Table 1).

**TABLE 1:** Testing of clinical samples by *S*. Abortusequi real-time PCR assay and culture method

Sample type	Real-time PCR result	Culture result		Detection rate (%)	PAP (95% CI)	NAP (95% CI)
		Positive	Negative			
Tissue	Positive	102	34	83.95	100.00 (96.37–100.00)	43.33 (31.57–55.90)
	Negative	0	26			
Plasma	Positive	2	0	1.04	100.00 (17.77–100.00)	100.00 (98.02–100.00)
	Negative	0	190			
Vaginal swab	Positive	7	41	25.81	100.00 (64.57–100.00)	77.09 (70.41–82.64)
	Negative	0	138			

In comparison with the data from the culture method, our real-time PCR assay achieved a positive agreement percentage (PAP) of 100.00% (95% CI, 96.37% to 100.00%) and a negative agreement percentage (NAP) of 43.33% (95% CI, 31.57% to 55.90%) for tissue samples, a PAP of 100.00% (95% CI, 17.77% to 100.00%) and a NAP of 100.00% (95% CI, 98.02% to 100.00%) for plasma samples, and a PAP of 100.00% (95% CI, 64.57% to 100.00%) and a NAP of 77.09% (95% CI, 70.41% to 82.64%) for vaginal swab samples (Table 1).

**FIG 3:**
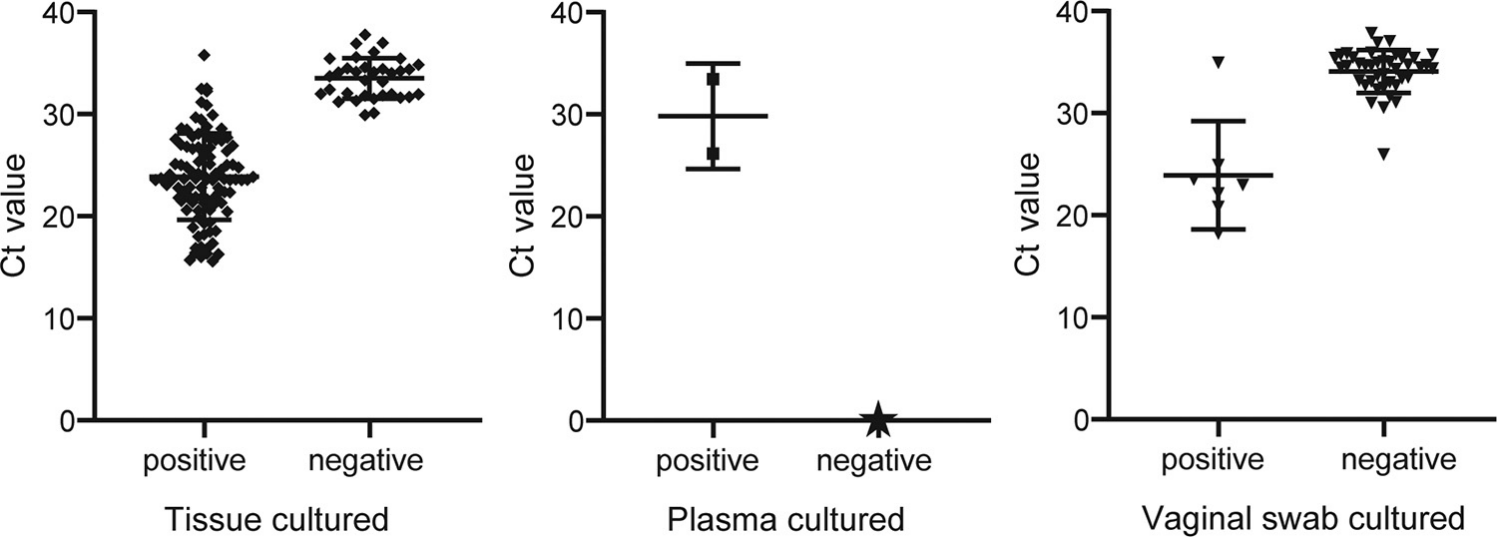
*C_T_* value distribution of positive samples identified by *S*. Abortusequi real-time PCR assay. The star symbol on the plasma scatter diagram is a meaningless value and was used to assist formation of an integrated picture.

Of the culture-positive clinical samples, the mean *C_T_* values were 23.89 ± 4.229, 29.81 ± 5.169, and 23.93 ± 5.302 for tissue, plasma, and vaginal swab samples, respectively. Of the culture-negative clinical samples, the mean *C_T_* values were 33.50 ± 1.579 and 34.09 ± 2.092 for tissue and vaginal swab samples, respectively (Fig. 3).

Comparing the data from our real-time PCR assay with that from the culture method, a ROC analysis was used to assess the performance of the *S*. Abortusequi real-time PCR assay in the detection of different types of samples. The area under the curve produced by the ROC analysis was 0.987 (*P* < 0.0001), 1.000 (*P* < 0.0001), and 0.979 (*P* < 0.0001) for tissue, plasma, and vaginal swab samples, respectively (Fig. S2). All results demonstrated that our assay was a reliable method to test for *S.* Abortusequi in tissue, plasma, and vaginal swab samples.

## DISCUSSION

A horse breeding farm in Inner Mongolia, North China, has reported that for the last 10 years, most of their mares have aborted late in pregnancy without any premonitory symptoms. *S.* Abortusequi was isolated by our laboratory from the organs of an aborted fetus from this farm. Recently, increasing numbers of horses and donkeys have been infected with Salmonella, with 30% to 100% morbidity on breeding farms (8). More surprisingly, according to our data, all Salmonella isolates in samples from equine abortions caused by Salmonella were identified as the *S.* Abortusequi serotype. Abortion in mares and donkeys caused by *S.* Abortusequi infection has resulted in significant economic losses. Conventional Salmonella detection methods involve culturing, which requires a series of different typing antisera to identify the serotypes involved (27). However, it takes time to order the antisera, and they are very expensive.

To the best of our knowledge, no molecular method capable of the specific identification of *S.* Abortusequi has been published to date. Several real-time PCR assays have been reported that target different genes (include *inv*, *fimY*, *siiA*, and others) are used for the detection of multiple subspecies of S. enterica (20, 28, 29). The full-length sequences of the genes encoding InvA, FljB, and OmpA were amplified from *S.* Abortusequi and sequenced. The resulting sequences were analyzed by BLAST online against National Center for Biotechnology Information database, and we found that the FljB gene can be considered a good candidate gene for the detection of *S*. Abortusequi. As shown in Fig. 1, the primers and probe sequences used in our assay could distinguish *S.* Abortusequi from *S.* Typhimurium, S. Enteritidis, *S.* Dublin, and most Salmonella serotypes from group A to group F. Although the PCR can also detect the FljB gene of seven strains from other Salmonella serovars in both the formula of H: e,n,x (*S*. Abony, *S*. Chester, and *S*. Bonariensis in group B, *S*. Gatuni in group C, and *S*. Rubislaw in group F) and that of H: e,n,z (*S*. Potsdam and *S*. Mikawasima in group C), these Salmonella serovars have never been reported to cause miscarriages in horses. Thus, in combination with clinical data, this real-time PCR would be very helpful for disease diagnosis.

In our experiments, the real-time PCR assay showed good specificity. The assay positively identified *S.* Abortusequi, whereas no amplification was obtained for other serovars of S. enterica, (*S.* Typhimurium, S. Enteritidis, and *S.* Dublin) that may also cause abortion in equines. Similarly, no amplification was obtained for seven other tested equine infectious disease pathogens.

Our assay also showed good sensitivity, with a 100% detection rate at concentrations of 30 copies/μL of the standard plasmid and 10 CFU/μL of bacterial DNA. The sensitivity of our assay was equal to that of other Salmonella real-time PCR assays (21, 30). The coefficients of intra-assay and interassay variation were satisfactorily low (less than 5%). In brief, the *S*. Abortusequi real-time PCR assay developed here was highly specific, sensitive, and reproducible.

Furthermore, we assessed the performance of the *S*. Abortusequi real-time PCR assay in 540 clinical samples. Our assay agreed 100% with the results of the culture method for culture-positive samples. Of the culture-negative samples, our assay tested 34 tissue samples and 41 vaginal swab samples as positive, meaning that our assay had a higher detection rate than did the culture method. One of the possible reasons for this is that the culture method only serves as a useful tool for the identification of live bacteria, while living or dead or impaired bacteria can be detected using *S.* Abortusequi real-time PCR. Another possible reason for the high detection rate of our assay is that we chose a reliable reagent and designed an effective set of primers and probe. On the other hand, our results had a lower positive detection rate, 25.8% (48/186), for vaginal swabs than for tissue samples, 84.0% (136/162). All tissue samples were collected from a farm where abortions occurred, whereas most vaginal swab samples were taken from equids on a healthy farm. In the tested vaginal swab samples collected from the farm where abortions occurred, the positive detection rate was 60% (26/40). This suggests that the vaginal swab could be used to confirm cases.

Using an IPC could help to monitor the presence of PCR inhibitors and confirm the efficiency of the sample preparation and extraction. We have tested to include a universal IPC (26) in our real-time PCR assay. We found that the amplification efficiency and slopes of standard curves for the assays were little different, with or without IPC (Fig. 2). Also, there was little difference in the *C_T_* values of IPC when testing 5 tissue samples obtained from aborted foals (Table S3). These results indicated that our samples received little interference from PCR inhibitors or extraction under laboratory conditions. Although adding the IPC could result in additional overhead costs, higher requirements for instruments, and increased workload, it is always good to add a universal IPC in a PCR assay to ensure the quantity of the sample preparation and extraction.

Taking the results together, the *S.* Abortusequi real-time PCR assay can reliably identify *S.* Abortusequi within 45 min, while the culture method normally takes at least 3 days to complete. The rapid identification of *S.* Abortusequi saves time and allows for the rapid implementation of clinical management.

In conclusion, we developed an effective *S.* Abortusequi real-time PCR assay and evaluated it by comparing it to the reference culture method. This assay could serve as a reliable tool for the rapid detection of *S*. Abortusequi in clinical tissue, plasma, and vaginal swab samples.
