# Supplementary material for: Development and Application of Real-Time PCR Assay for Detection of Salmonella Abortusequi
Source: J Clin Microbiol. 2023 Mar 1;61(3):e01375-22. doi: 10.1128/jcm.01375-22 (PMC10035326; doi:10.1128/jcm.01375-22)
Supplement: Supplemental file 1 — Supplemental material. Download jcm.01375-22-s0001.pdf, PDF file, 1.1 MB [file jcm.01375-22-s0001.pdf]

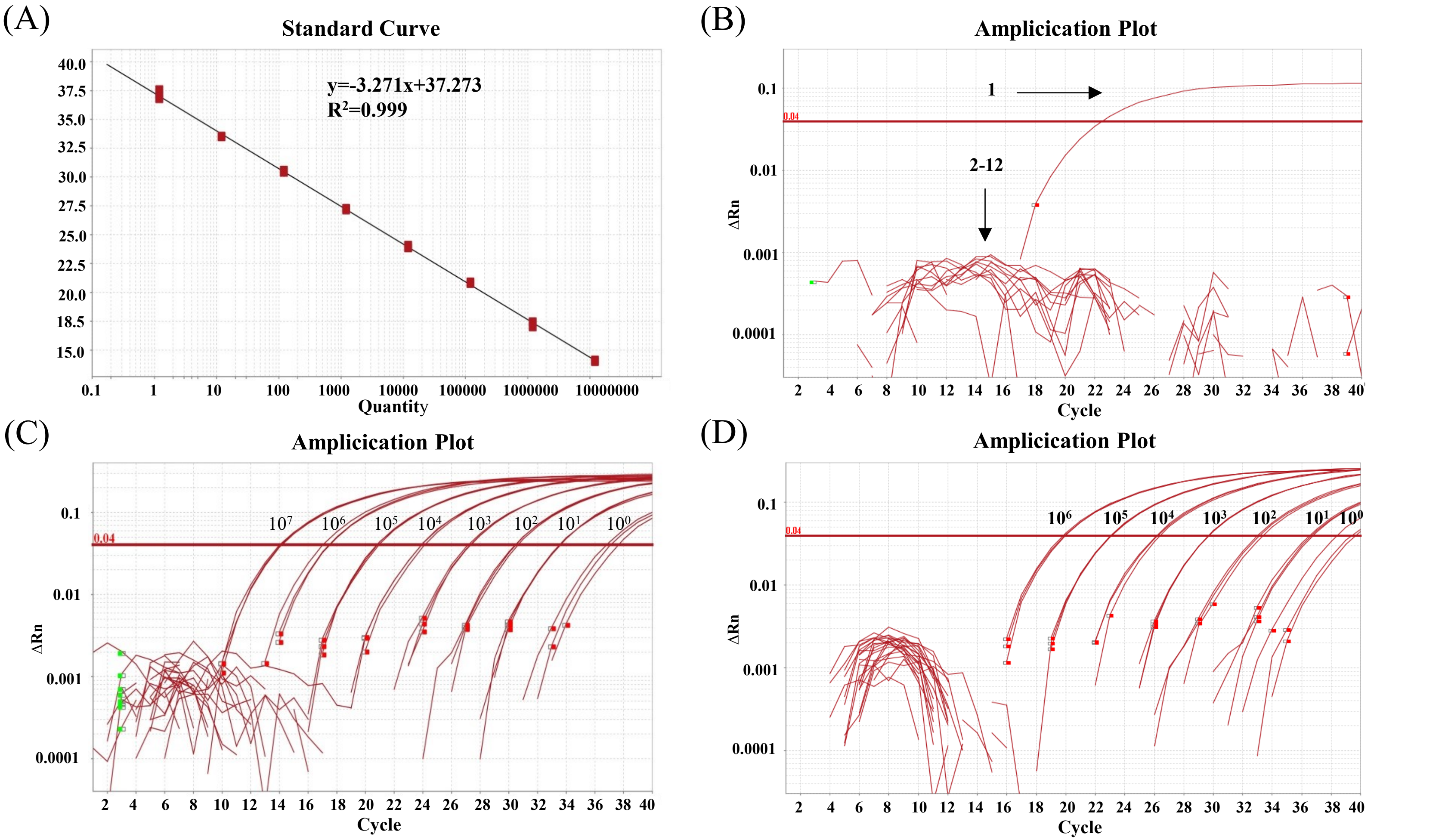

FIG S1 Evaluation of the *S.* Abortusequi real time PCR assay. (A) The standard curve of the *S.* Abortusequi real time PCR assay. (B) The specificity of the *S.* Abortusequi real time PCR assay. Curves 1-12, nucleic acid templates corresponding to *S.* Abortusequi, *S. equi*, EIV, EHV-1, EHV-4, EIAV, EAV, *E. coli*, *S.* Typhimurium, *S.* Enteritidis, *S.* Dublin, and negative control. (C) The sensitivity of the *S.* Abortusequi real time PCR assay for testing standard plasmid ranging from 3×10^7 to 3×10^0 copies/μL from left to right. (D) The sensitivity of the *S.* Abortusequi real time PCR assay for testing bacteria DNA ranging from 1×10^6 to 1×10^0 CFU/μL from left to right.

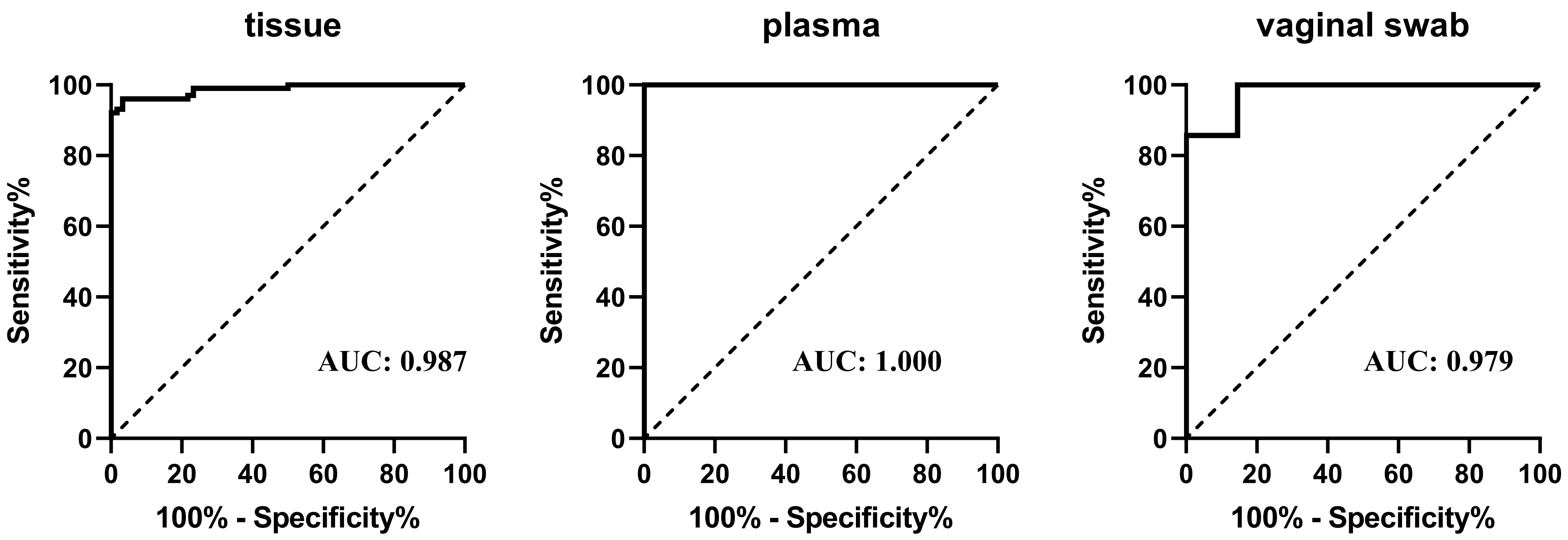

FIG S2 ROC curves of the *S.* Abortusequi real time PCR assay for tissue, plasma, and vaginal swab samples.

TABLE S1 Confirmation of the sensitivity

Plasmid			Bacteria		
Concentration (copies/μL)	No. tested	No. positive	Concentration (CFU/μL)	No. tested	No. positive
3×10^2	10	10	1×10^2	10	10
3×10^1	10	10	1×10^1	10	10
3×10^0	10	8	1×10^0	10	3

TABLE S2 Reproducibility of the *S.* Abortusequi real time PCR assay. (A) The mean±standard deviation (M ± SD) cycle threshold (Ct) value and coefficient of variation (CV) of intra-assay. (B) The M ± SD Ct value and CV of inter-assay

(A)

Plasmid			Bacteria		
Concentration (copies/μL)	M ± SD Ct value	CV (%)	Concentration (CFU/μL)	M ± SD Ct value	CV (%)
3×10^5	20.83 ± 0.06	0.30	1×10^5	23.08 ± 0.02	0.09
3×10^4	23.95 ± 0.11	0.45	1×10^4	26.24 ± 0.10	0.40
3×10^3	27.24 ± 0.06	0.24	1×10^3	29.68 ± 0.01	0.05
3×10^2	30.48 ± 0.09	0.29	1×10^2	33.18 ± 0.16	0.47
3×10^1	33.52 ± 0.03	0.08	1×10^1	36.64 ± 0.11	0.30
3×10^0	37.14 ± 0.33	0.88	1×10^0	39.14 ± 0.55	1.40

(B)

Plasmid			Bacteria		
Concentration (copies/μL)	M ± SD Ct value	CV (%)	Concentration (CFU /μL)	M ± SD Ct value	CV (%)
3×10^5	20.90 ± 0.15	0.69	1×10^5	21.79 ± 0.94	4.31
3×10^4	24.33 ± 0.32	1.33	1×10^4	25.35 ± 0.66	2.59
3×10^3	27.76 ± 0.38	1.38	1×10^3	28.87 ± 0.59	2.06
3×10^2	31.02 ± 0.39	1.26	1×10^2	32.87 ± 0.61	1.87
3×10^1	34.35 ± 0.65	1.89	1×10^1	36.08 ± 0.68	1.89
3×10^0	37.66 ± 0.58	1.55	1×10^0	38.54 ± 0.65	1.68

TABLE S3 Real time PCRs with IPC addition

Tissue samples	*S.* Abortusequi real time PCR with IPC addition				Real time PCR with IPC alone		IPC CV (%)
	S. Abortusequi (Ct)		IPC (Ct)		IPC		
Heart	24.48	24.67	30.33	30.22	30.38	30.59	
Liver	21.26	21.39	30.47	30.51	30.52	30.66	
Spleen	20.38	20.68	30.87	31.09	31.04	30.30	1.26
Lung	22.43	22.36	31.53	30.46	30.50	31.06	
Kidney	23.34	23.32	31.52	30.39	30.38	30.59	
